# Supplementary material for: Identification of photoperiod-induced specific miRNAs in the adrenal glands of Sunite sheep (Ovis aries)
Source: Front Vet Sci. 2022 Jul 22;9:888207. doi: 10.3389/fvets.2022.888207 (PMC9354845; doi:10.3389/fvets.2022.888207)
Supplement: Supplementary file 1 [file Data_Sheet_1.ZIP › Supplementary file/miRDeep2 file.zip!/miRDeep2 file/Know_Structure/SPLP42A1/oar-mir-376b.pdf]

miRBase precursor : oar-mir-376b
Total read count : 511
oar-miR-376e-5p read count : 200
oar-miR-376b-5p read count : 200
oar-miR-376b-3p read count : 110
remaining reads : 1

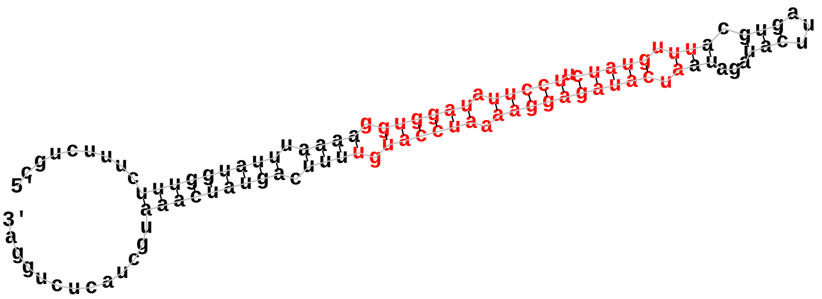

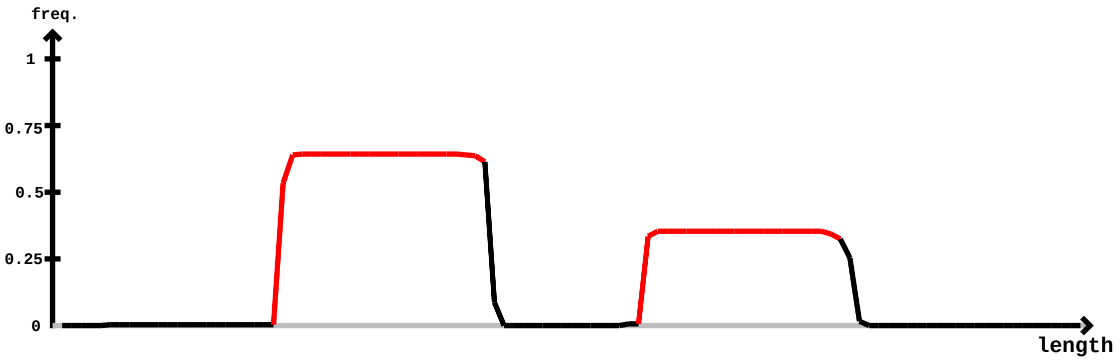

oar-miR-376b-3p
oar-miR-376b-5p
oar-miR-376e-5p

```
5'- cgucuuucuuugguauuuaaaagguggauauuccuucuauguuuacgugauucauagauaaucauagaggaaaauccauguuuucaguaucaaaugcuacucugga  -3'    exp
    ........((((((((.((((.((((((.(((((.(((((.(((.(((...)))...))).)))))))))).)))))).)))).))))))))..............     reads   mm    sample
    ....uuucuuugguauuuaaaag...................................................................................     1       0     seq
    ......................gguggauauuccuucuaug.................................................................     1       0     seq
    ......................gguggauauuccuucuauguu...............................................................     5       0     seq
    ......................gguggauauuccuucuauguuu..............................................................     135     0     seq
    ......................gguggauauuccuucuauguuua.............................................................     24      0     seq
    .......................guggauauuccuucuaugu................................................................     1       0     seq
    .......................guggauauuccuucuauguu...............................................................     2       0     seq
    .......................guggauauuccuucuauguuu..............................................................     29      0     seq
    .......................guggauauuccuucuauguuua.............................................................     2       0     seq
    ........................uggauauuccuucuauguuua.............................................................     1       0     seq
    ..........................................................uaaucauagaggaaaauccaug..........................     2       0     seq
    ............................................................aucauagaggaaaauccau...........................     3       0     seq
    ............................................................aucauagaggaaaauccaug..........................     4       0     seq
    ............................................................aucauagaggaaaauccaugu.........................     21      0     seq
    ............................................................aucauagaggaaaauccauguu........................     72      0     seq
    ............................................................aucauagaggaaaauccauguuu.......................     2       0     seq
    .............................................................ucauagaggaaaauccaugu.........................     1       0     seq
    .............................................................ucauagaggaaaauccauguu........................     2       0     seq
    .............................................................ucauagaggaaaauccauguuu.......................     3       0     seq
```
